# Case Report: Disseminated Cysticercosis due to Intentional Ingestion of Parasitic Worm Eggs for Weight Loss

**DOI:** 10.4269/ajtmh.21-0760

**Published:** 2021-11-29

**Authors:** Han-Yu Zhang, Guo-Xing Wang, Yue-Yan Xing, Miao-Rong Xie

**Affiliations:** Emergency Department, Beijing Friendship Hospital, Capital Medical University, Beijing, China

## Abstract

A 20-year-old female resident of Beijing intended to consume the eggs of the parasitic worm, *Taenia saginata*, for weight loss; however, she apparently inadvertently ingested *Taenia solium* (pork tapeworm) eggs, which resulted in disseminated cysticercosis. Cysticerci developed in the brain, tongue, muscles, liver, peritoneum, and subcutaneous tissues. She was administered oral albendazole and praziquantel. After four 10-day courses of treatment, most of the cysts disappeared and she recovered. After 3 years, the patient remains in good health.

Cysticercosis is a health problem in most developing countries,^1^ but it is rare in modern cities like Beijing. Moreover, extensive disseminated cysticercosis is particularly uncommon because of improved hygiene. Although over 100 cases of disseminated cysticercosis have been reported, there is no specific report of the inappropriate ingestion of *Taenia solium* for weight loss in the full literature. We report a case of disseminated cysticercosis in a 20-year-old Beijing woman who intended to consume the eggs of the parasitic worm, *Taenia saginata*, to induce weight loss, but mistakenly ingested *T. solium eggs*.

## CASE PRESENTATION

On March 2, 2018, an obese (110 kg, body mass index [BMI] 38.1 kg/m^2^) 20-year-old female resident of Beijing was admitted to the Emergency Department of Beijing Friendship Hospital (Capital Medical University, Beijing, China) with chronic headache and abdominal pain.

The patient reported that she weighed 150 kg in April 2017 and was concerned about her weight. After reading online that consuming parasitic worm eggs could induce weight loss, she purchased “*T. saginata* eggs” in capsules and consumed two capsules around May 27, 2017. She lost 27 kg in the following 4 months; however, because she remained concerned about her weight, she consumed another capsule around September 30, 2017.

**Figure 1.**
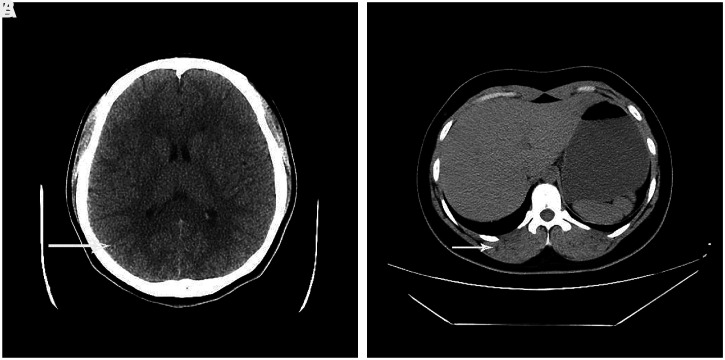
(**A** and** B**) Computed tomography scans of the head and abdomen (November 2017) reveal multiple, small low-density lesions in brain parenchyma and muscle.

She was admitted to our hospital because of complaints of headache and nausea on November 2, 2017. “No abnormalities” were detected on a computed tomography (CT) scan of her head and abdomen (in fact, there was something abnormal on the CT, but we did not note it) (Figure 1A and B). The cerebrospinal fluid (CSF) pressure was > 300 mmH_2_O (normal: 80–180 mmH_2_O), but no abnormalities were detected on cytological, chemical, and microbiological CSF examinations. The stool tests revealed normal findings. She declined magnetic resonance imaging (MRI) for financial reasons. She was diagnosed with viral encephalitis and cranial hypertension. Her symptoms resolved after treatment with acyclovir and mannitol injections, and she was discharged on November 16, 2017. She continued to experience occasional dull headaches.

On March 2, 2018, she was readmitted because of severe abdominal pain and headache without fever, blurred vision, or vomiting. She reported no membranous discharge in her stool.

On examination, multiple, soft-to-firm, deep-seated asymptomatic nodular lesions were detected on her torso, head, and neck. No other physical abnormalities were detected.

**Figure 2.**
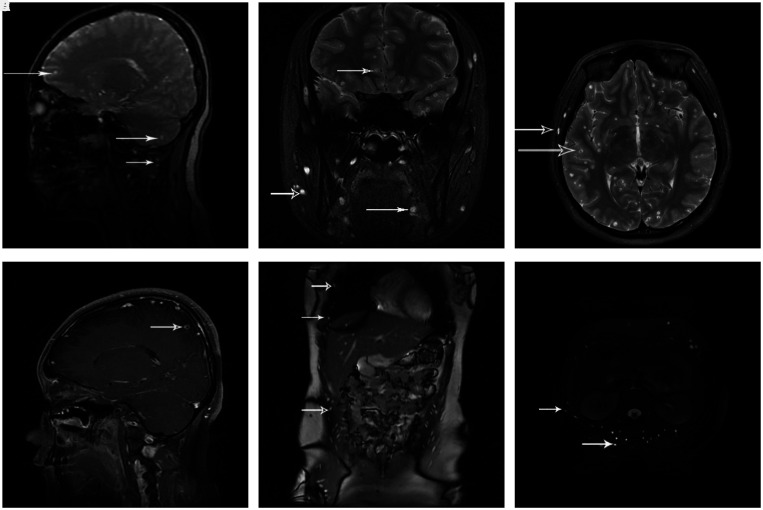
(**A**,** B**,** C**, and** D**) Magnetic resonance imaging of the head (March 2018) reveals multiple cystic lesions in the bilateral cerebral cortex, parenchyma and cerebellum, subcutaneous tissues, and muscles of the face, neck, and tongue. (**E** and** F**) Magnetic resonance imaging of the abdomen (March 2018) reveals cystic lesions in the abdominal muscles, liver, peritoneum, and erector spinae muscle.

Stool examination revealed normal findings, with no eggs or gravid proglottids. An enzyme-linked immunosorbent assay for serum cysticercosis antibodies was positive. Head MRI revealed multiple cystic lesions in the bilateral cerebral cortex, bilateral parenchyma and cerebellum, subcutaneous tissues, and muscles of the face, neck, and tongue (Figure 2A–D). Abdominal MRI revealed cystic lesions in the abdominal muscles, liver, and peritoneum (Figure 2E and F). No abnormality was detected in her CSF, except for CSF pressure > 320 mmH_2_O. An electroencephalogram revealed slow, high-amplitude waves throughout the cortex.

The patient was diagnosed with severe disseminated cysticercosis affecting the brain, tongue, muscles, liver, peritoneum, and subcutaneous tissues. The infection was complicated by cranial hypertension and latent epilepsy.

The patient reported that she had never eaten raw meat and that she maintains good personal hygiene. None of her family members or coworkers had known cysticercosis or tapeworm infestations, and there was no report of infestation in her community. We speculated that the “*T. saginata* eggs” that she had consumed for weight loss in 2017 were in fact *T. solium* eggs.

**Figure 3.**
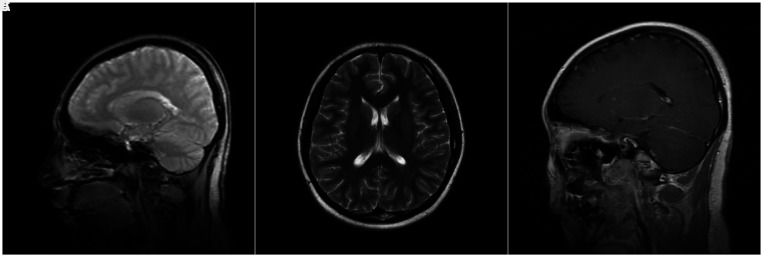
(**A**,** B**, and** C**) Magnetic resonance imaging of the head (August 2018) reveals that cystic granulomas almost disappeared after four courses of treatment.

Based on the treatment protocol for neurocysticercosis,^2^ she was administered oral albendazole (15 mg/kg/day) and praziquantel (50 mg/kg/day) twice daily with 5 mg dexamethasone daily for 10 days and then suspended for 20 days. After four courses of albendazole and praziquantel, the cysts almost entirely disappeared (Figure 3A–C).

**Figure 4.**
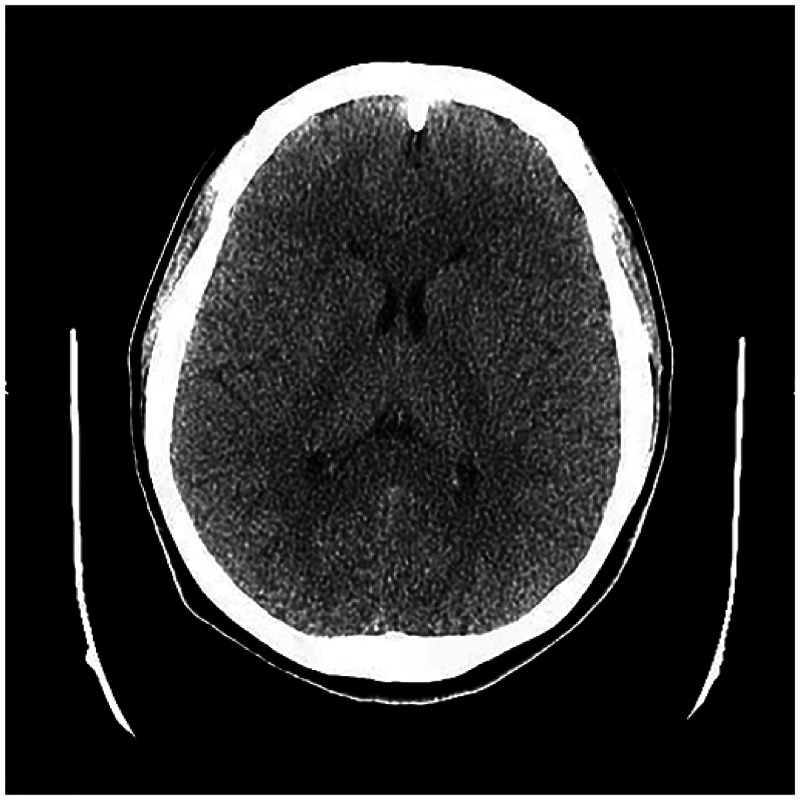
Computed tomography scans of the head (March 2021) reveal no abnormalities.

At the most recent follow-up (March 2021), the patient was in good health and weighed 75 kg, with a BMI of 26.0 kg/m^2^, and there was no evidence of cysticercosis nodules (Figure 4).

## DISCUSSION

A literature search using Medline and Embase revealed that although over 100 cases of disseminated cysticercosis have been reported, there are no specific reports of the inappropriate ingestion of *T. solium* for weight loss in a full literature. Therefore, this report provides new and valuable information on cysticercosis.

Cysticercosis remains a serious public health problem, especially in developing countries. The World Health Organization lists cysticercosis as a marginalized disease that requires a comprehensive approach.^3^ The incidence of cysticercosis in China has decreased significantly as a result of the reduction of pork, eggs, and water contaminated with *T. solium* eggs;^4^ however, cysticercosis caused by the inappropriate ingestion of *T. solium* eggs has been reported sketchily.^5^^,^^6^

Ingestion of *T. solium* eggs or fecal-oral auto-transmission in individuals harboring intestinal cestodes are two main sources through which humans contact the causative agent of cysticercosis.^7^ *Taenia solium* eggs develop into oncospheres that penetrate the gut wall, enter the blood and lymphatic vessels, and then penetrate various tissues and organs, where they develop into cysticerci that cause the clinical symptoms of cysticercosis.^8^

Humans can be the intermediate or final host of *T. solium* and thus can develop intestinal taeniasis or cysticercosis.^8^^,^^9^ Conversely, humans cannot be the intermediate host of *T. saginata* because the human body has natural immunity to the larvae of *T. saginata*, so human cysticercosis does not occur.^10^ Cysticercosis is caused by the ingestion of *T. solium* eggs shed in the stool of a human tapeworm carrier.^2^ The patient had no history of consuming undercooked pork or contact with other human cases; therefore, we speculate that she inadvertently consumed *T. solium* eggs assuming that they were *T. saginata* eggs.

Parasitologist Kozen Yoshino and three volunteers made a significant contribution to current knowledge by intentionally ingesting cysticerci to describe human *T. solium* infection. Between 62 and 72 days after ingesting the cysticerci, gravid proglottids were detected in their feces.^11^^–^^14^ None of them developed cysticercosis. Previous studies reported that it took approximately 5 months for tapeworm eggs to develop into cysticercosis in *SCID* mice^15^ and approximately 6 months in rhesus monkeys.^16^ Studies among immigrants to the United States have found that neurocysticercosis usually becomes symptomatic 3–5 years after infestation,^17^ and the median time for the diagnosis of cysticercosis is approximately 58 months.^18^ To our knowledge, only two cases of inadvertent ingestion of tapeworm eggs (one of whom was also admitted to our hospital) have been reported. Neither patient provided a specific date of ingestion.^5^^,^^6^ Our patient ingested the eggs in May and September 2017, and developed headache, nausea, and high intracranial pressure in November 2017. We identified multiple small low-density lesions on CT from November 2017, and an MRI revealed cysticercosis in March 2018. She was diagnosed with cysticercosis 10 months after ingesting the eggs. The incubation period for tapeworm eggs to develop into cysticercosis was 2–10 months in our patient. We speculate that the incubation period may have been 2–6 months, because she developed symptoms 6 months after consuming the capsules, and she may have had cysticercosis at the time of her first admission. She was not diagnosed with cysticercosis at that time because she did not indicate that she had ingested tapeworm eggs. Moreover, no eggs were found in her stool, no cysticercosis test was performed. There was something abnormal on the first CT, but we did not note it, and she refused to undergo MRI. To our knowledge, this report provides the most accurate estimate available of the incubation period of tapeworm eggs to develop into cysticerci in the human body.

The clinical presentation of cysticercosis depends on the site, size, number of lesions, and host inflammatory response.^2^ The number of lesions is related to the number of eggs ingested and the host’s immunity status.^16^^,^^19^ Immune response plays an important role in the relationship between the host and cysticerci.^19^ If a small number of cysticerci invade an individual, the host immune system can eradicate them within a short period; however, when a large number of eggs is ingested, disseminated cysticercosis could result.^9^^,^^20^ To survive, the parasite must evade the host’s immune surveillance, consume nutrients,^21^ and even adjust the physiology of the host to suit its needs.^22^ Disseminated cysticercosis usually occurs in immunocompromised individuals.^23^ Parasites at different stages of development, including active, transitional, and inactive forms, may coexist.^24^ If all parasites are at the same stage of development, it may indicate that the host’s immune system is naïve to the parasite and many eggs were ingested simultaneously.^25^^,^^26^

As shown in this report, deliberate parasitic infection for weight loss is dangerous. Clinicians should be aware of all forms of parasitic infection and modes of transmission.
